# Southern Dental Association

**Published:** 1886-10

**Authors:** 


### SOUTHERN DENTAL ASSOCIATION.

EIGHTEENTH ANNUAL MEETING, HELD AT NASHVILLE, TENN., JULY 27, 28, 29 AND 30, 1886.

REPORTED FOR THE INDEPENDENT PRACTITIONER BY "MRS. M. W. J."

(Concluded from page 505.)

Dr. B. H. Teague read a paper entitled

### THE PERSONAL HYGIENE OF THE DENTIST.

He portrayed the health-giving conditions of early practitioners, when itineracy was the rule, and the dentist had the healthy muscular exercise of hand-pressure in operating and the rolling-mill in the laboratory, contrasting it with the present sedentary life of the city dentist. The older generation of dentists are, as a rule, robust, hearty old men, while the younger ones are pale and slender, subject to ailments of stomach, liver, lungs and nerves, the former aggravated by blue pills and liver regulators, the latter by strong cigars; rest sought only on the lounge while reading the journals, and sleep sacrificed to recording incidents or composing articles. The remedy for all this should be outdoor exercise, careful diet, absolute cleanliness and the aid of various appliances, as the armrest, the operating stool, mechanical motors, systematic appointments and short sittings, care of the eyes, and a summer vacation.

This very practical paper was discussed at some length, with widely varying opinions as to what constitutes the best diet and the best light.

Dr. Wm. Crenshaw read a paper entitled

A COMPLETE SYSTEM POSSIBLE IN FILLING TEETH.

His idea of a perfect system was embraced in the exclusive use of the electric mallet, Perry's separators, the rubber dam and cohesive gold for every operation, restoring the correct anatomical form of every tooth, which, if naturally below the ideal, should be cut and trimmed and the surface restored in gold. When material is varied, changed or abandoned with every variation of circumstances, systematic work is impossible. He considered matrices a snare, and hand and automatic mallets risky devices. Only with the electric mallet and cohesive gold can uniformly perfect, systematic work be done.

Dr. Salomon read a paper from Dr. Knapp on

NERVE (PULP?) CANALS.

He considered that the probabilities were largely against success in attempts to save the pulps when once exposed by caries, it being, as a general rule, admitted that exposure of the pulp is soon followed by loss of vitality, the best operators now removing it entirely, filling the canal to the foramen. To facilitate the removal of the pulp the crown may be much cut away and restored by filling, the pulp canal being made funnel-shaped, to facilitate the passage of instruments and the removal of the contents. With the aid of a delicately pointed stick of osier or orange wood, placed at the entrance of the canal and tapped two or three times with the mallet, the pulp is more readily removed than with the barbed broach. The canal can be filled with gold, lead, oxychloride or oxyphosphate of zinc, or gutta-percha dissolved in chloroform, the writer preferring wood trimmed to fit, and dipped in carbolic acid.

These papers were discussed at some length, neither of them finding advocates of the extreme views taken, the general voice being in favor of combining the best methods of the best operators, and selecting the materials and means best adapted to each case as it presents itself. The members also favored the preservation of the pulp whenever possible.

Dr. E. S. Chisholm read a paper on

HISTOLOGY AND MICROSCOPY.

To understand the etiology of the decay of the human teeth implies the ability to differentiate between physiological and pathological conditions, the microscope giving the proof of ocular demonstration of histological conditions; hence the value of these studies to the dental practitioner. The microscope reveals the presence of bacteria and other organisms, but it cannot demonstrate the part they play in the destruction of the teeth. Demonstration is the witness, reason the judge; the verdict is science.

This paper and subject passed without discussion.

PYORRHŒA ALVEOLARIS.

Dr. R. B. Adair gave an oral explanation of his very successful treatment, not having been able to find a patient on whom to demonstrate the action of his remedies.

He said that surgical treatment, though very essential in its place, would not restore lost tissues; the pockets are still there, and food crammed into them keeps up the irritation and prevents granulation. The second step is to thoroughly protect the parts that have first been thoroughly cleansed from all deposits and debris. Plates and sponge grafts and other devices often fail, but with the following remedies he has been uniformly successful in inducing healthy granulation, and protecting the parts until the lost tissues are replaced, the teeth tightened and the gum restored. He has no failures to record.

The preparations are as follows :

1. Crystals of Iodine are dissolved in enough pure wood creosote to make a saturated solution (which improves with age). This is applied down in the pockets, and all over the suppurating surface, destroying germs and stimulating healthy granulation.

2. A glycerole of tannin is made by packing in a small wide-mouthed bottle as much crystals of tannin as it will hold, adding enough glycerine to dissolve it, making a very thick solution. After the application of the first remedy the surfaces are dried and protected from saliva, and the second remedy applied. The overflow of saliva coming in contact, forms a pellicle which resists friction, sealing up the pockets and protecting them properly. The patient sbould be instructed not to disturb this. The next day this should be removed, coming off like the inner skin of an egg-shell, and the applications should be renewed. Healthy granulation takes place, and the pockets fill up with new tissue in a period varying from ten to a hundred days.

*Dr. Catching*—Vouched for the virtue of Dr. Adair's mode of treatment. He believes there is always caries of the margin of the process, which he trims away thoroughly, treating with sulphuric acid, followed by iodine, or chloride of zinc forty grains to the ounce, which he applies with a sable hair brush, having a labeled brush for each patient.

*Dr. G. Chisholm*—Uses Dr. Adair's remedies, supplemented with the following mouth-wash :

| | |
|---|---|
| Crystals Iodine, | 3 gr. |
| Tincture Aconite, | 3 drachms. |
| Myrrh | 1 oz. |
| Tannin, | 10 gr. |
| Gaultheria, | q. s. |

Alcohol sufficient to make 3 oz.

He finds it difficult to secure the return of patients regularly, and this keeps the mouth in good condition, preventing the formation of pus between sittings.

*Dr. Morgan*—Said that any treatment that was not based on a knowledge of the cause of disease was empirical and quackish.

*Dr. Crawford*—Questioned whether the disease might not have its origin in the cementum, or the pericementum, the latter being the first structure to make outcry. If it depends on constitutional conditions we must go beyond local treatment, which in that case can only be palliative.

*Dr. Thackston*—Spoke of the pathology of half a century ago, and the teachings of Horace Hayden. This identical disease was as well known then as now, and was called "conjoined suppuration of the gums and alveolar processes." It was considered the local exhibition of constitutional disease. It was then believed that there was no cure for it, the treatment embracing a building up of the whole body, to hold in abeyance the ravages of a disease for which there was no radical cure. The success of modern systems of treatment is far in advance of any they achieved in that day, especially through surgical treatment in the removal of deposits and debris.

*Dr. J. Taft*—Condemned the nomenclature of to-day, names which were not appropriate becoming popular or fashionable. The old descriptive names recalled by Dr. Thackston were, perhaps, as as good as any.

He spoke in detail of the various stages and phases of the disease, systemic conditions and local irritants, microbes, etc., and the mode of treatment based upon a knowledge of these conditions. Know first what you are aiming at, and what you have to accomplish, and what to overcome, and then select the remedies best adapted to those wants, as the carpenter selects the tool suited to his purpose. Peroxide of hydrogen is a grand agent, preventing decomposition, destroying and eliminating organisms, allowing the surfaces to heal properly.

*Dr. Thackston*—Spoke of the reddish-brown deposits enveloping the apex of roots of teeth around which the gums are firmly attached; deposits evidently not from the saliva, nor from the gums, but from the blood itself.

*Dr. Taft*—Thought these deposits extremely rare, unless there had been an alveolar abscess; not ordinary, but extreme cases.

*Dr. Rembert*—Defined serumal calculus as a product of pus-flow, degenerated plasm thrown out, with calcific precipitation deposited from serum or decomposing blood exuding through the tissues.

*Dr. J. J. R. Patrick*—Said that chemical analysis had never shown any distinction between sanguinary and salivary calculus, the only difference lying in the mode of deposit; salivary calculus is a secretion, sanguinary calculus a concretion.

*Dr. Morgan*—Thought the only radical cure for this disease was the removal of the teeth, though if the cementum is entirely removed by scraping, and the root made perfectly smooth and polished, the gums heal up. The trouble, therefore, possibly lies in the cementum, or the periosteum, except where hereditary. In the latter case, if taken in hand early, in young children, the application of cotton saturated with carbolic acid or iodine, will, with the aid of constitutional treatment, sometimes relieve the ichorous discharge.

The dark deposits are the results of the decomposition of the tissues of the blood. Lime-salts are carried in the blood, and in the breaking up of the blood tissues the lime-salts have to be disposed of, and they crystallize about the roots of the tooth. In his experience of forty years he had not seen more than half a dozen cases where there was necrosed alveolar process. The bone is softened, the lime-salts being dissolved and carried off in the general circulation, but this is a physiological process, and never takes place with dead tissues. The surgical operation of removing deposits and debris should be thorough at the first sitting, when the soft tissues are broken up and blood poured out, forming a fibrous coagulum which should not be disturbed. The pockets fill up, but the tissue formed is of the nature of scar tissue or cartilage, holding the teeth firmly in place, but the periosteum is never reproduced. Robinson's remedy is very valuable for stimulating granulation, and diet is an important ally in the treatment.

*Dr. J. H. Moore*—Said that his observations led him to the conclusion that pyorrhœa was always connected with catarrh, particularly that form in which there are no deposits on the roots of the teeth, but deep pockets formed, with exudations of pus, and all the other symptoms of the disease.

### FRIDAY MORNING SESSION.

Drs. Thackston, J. Hall Moore, and G. H. Winkler were appointed a committee to investigate resolutions of impeachment, or charges of ungentlemanly and unprofessional conduct, brought against a member of the Association.

A committee was also appointed to draft resolutions on the death of Drs. Holmes, Best, Jobson and Redman.

Resolutions of thanks were passed to the officers of Watkins' Institute, the faculty of Vanderbilt University, the Y. M. C. A., the Tennessee Historical Society, and the Nashville Art Association.

As the report of the Committee on Chemistry, Dr. Theo. Johnston, read a paper entitled

### GOLD—COHESIVE AND NON-COHESIVE.

He thought the principles of chemistry involved in the preparation of gold for dental purposes should be taught in every dental college. If we understood clearly why our gold is or is not cohesive, we should have less trouble in handling it. He considered the term "cohesive," as used by the practitioner, a misnomer, the pieces of gold in a filling being really welded. He reviewed in detail the great variety of properties possessed by gold, in which it surpasses all other metals; defined the principles of chemistry involved in the preparation of gold for the dentists' uses, claiming that absolute purity was essential for welding.

This latter point was not agreed to in the discussion which followed, Abbey's gold being admittedly alloyed with six per cent. silver, as pointed out by Dr. Morgan. Dr. Salomon showed that the addition of platinum to the extent of three per cent. to six per cent. does not prevent it from being very cohesive and easily worked.

The question of the union of gold and platinum being raised, Dr. Winkler said that if molten gold is stirred with a platinum wire, molecules of the latter float off, and being smaller than the molecules of gold, flow in between the latter, the two metals being thus mixed in cooling. Platinum-gold is of beautiful color for front fillings, being scarcely perceptible, even though one-third of an incisor be built down with it.

At the request of Dr. Crenshaw, the interrupted discussion of Operative Dentistry was resumed, that he might reply to criticisms of his paper.

The discussion was continued by Drs. Beech, Winkler, McKellops, Staples, Marshall, Freeman, Louis Chisholm and Crenshaw.

Dr. Catching related a well-authenticated case of remarkable tooth development. A child, born in 1871, began teething at the age of six months, erupting a full set of very small teeth, which were all shed within three months. At the age of eleven months she began teething again, and at the age of fifteen months had a second full set. These also soon crumbled away like chalk. When two years and a half old she weighed ten pounds, and had a third set of teeth. Suffering much from them, they were all extracted before she was four years old, but at seven years of age she had four front teeth of the fourth set. These were mere shells, and were picked off from the gum with the finger nails.

She began cutting a fifth set of teeth at the age of eleven, which she still retains, being now fifteen years of age. She is now a stout, healthy girl, budding into young womanhood; is a resident of Atlanta, Ga., and a patient of Dr. Catching.

Old Point Comfort, Va., was selected as the next place of meeting, and the following officers elected :

*President*—Dr. W. H. H. Thackston, Farmville, Va.
*First Vice-President*—Dr. B. H. Catching, Atlanta, Ga.
*Second Vice-President*—Dr. J. R. Knapp, New Orleans, La.
*Third Vice-President*—Dr. W. H. Richards, Knoxville, Tenn.
*Recording Secretary*—Dr. L. B. Dotterer, ———— S. Carolina.
*Corresponding Secretary*—Dr. J. I. Crawford, Nashville, Tenn.
*Treasurer*—Dr. H. A. Lowrance, Athens, Ga.

After the installation of the officers elect, the Association adjourned to meet on Monday, at the office of Dr. Morgan, for clinics, after which the Society adjourned finally.

The selection of the time of meeting was left to the Executive Committee, to be announced in due time.

